# Case Report: Marjolin ulcer characterized by squamous cell carcinoma arising from chronic wounds: clinical features, diagnostic and therapeutic challenges, and insights—a report of four cases

**DOI:** 10.3389/fmed.2026.1838998

**Published:** 2026-06-11

**Authors:** Lianbo Yang, Xinming Wang, Wanchen Zhou, Jingyi Cui, Lili Niu, Mingyu Hu, Qi Liu, Haidong Liang

**Affiliations:** Department of Orthopedic and Soft Tissue Reconstruction Surgery, The Second Hospital of Dalian Medical University, Dalian, Liaoning, China

**Keywords:** chronic trauma, cutaneous squamous cell carcinoma, diagnostic delay, Marjolin ulcer, warning signals

## Abstract

**Purpose:**

This study aimed to analyze the clinical features and diagnostic challenges of cutaneous squamous cell carcinoma arising from chronic limb wounds—a condition known as a Marjolin ulcer—to summarize practical warning signals for early recognition and to explore the underlying mechanism of malignant transformation associated with chronic inflammation.

**Methods:**

We present a detailed report of four elderly male patients. All of them had long-standing, non-healing chronic wounds on the limbs, with initial triggers including an ingrown nail, a foreign body injury, a post-surgical scar from a calcaneal fracture, and an abrasion. The wound duration ranged from 8 months to 30 years. Clinical presentations, imaging findings, surgical procedures, pathological results, and short-term postoperative outcomes were systematically reviewed.

**Results:**

All patients were initially misdiagnosed with benign chronic wounds, leading to diagnostic delays from months to decades. Final surgical pathology confirmed cutaneous squamous cell carcinoma in every case, with bone invasion present in two cases. Owing to these delays, treatment escalated from potentially curative local excision to amputation in three of the four cases. No local recurrence was detected during the short-term follow-up period.

**Conclusion:**

Based on these cases, we propose a practical set of clinical warning signals centered on wound chronicity, abnormal morphology, and patient demographics. Long-standing, non-healing wounds on the limbs of elderly male patients should prompt suspicion of malignant transformation. Early biopsy is essential to avoid devastating outcomes such as amputation. The chronic inflammatory microenvironment appears to serve as a continuous driving force behind this cancer development pathway.

## Introduction

Cutaneous squamous cell carcinoma (cSCC) is the second most common non-melanoma skin cancer worldwide, with an age-standardized incidence rate of 236.91 per 100,000 population. It accounts for approximately 20% of keratinocyte carcinoma diagnoses (1, 2). Generally, cSCC has a good prognosis; however, when it arises in the context of a chronic wound, a condition known as a Marjolin ulcer, the prognosis can be poor.

The pathogenesis of cSCC involves a complex interplay between environmental and genetic factors. Ultraviolet (UV) radiation is the predominant environmental driver, causing cumulative DNA damage (3). At the genetic level, key driver mutations in TP53, NOTCH, CDKN2A, and EGFR disrupt cell cycle control and squamous differentiation (4). Immunosuppression, whether drug-induced or disease-related, further elevates the risk (3, 4). These insights provide a framework for understanding how the chronic wound microenvironment—via persistent inflammation and impaired immune surveillance—may serve as an alternative carcinogenic pathway distinct from ultraviolet (UV)-driven cSCC. The majority of primary cSCCs (80–90%) occur in the head-and-neck region, are driven by ultraviolet radiation, and are treated surgically, with clear margins as the goal (5–7).

However, the four cases in this report are distinct. They are all caused by long-standing, chronic wounds or irritations on the limbs—including ingrown nails, hangnails, abrasions, and a post-fracture ulcer—fundamentally differing in etiology and clinical presentation from UV-driven cSCC (1, 2). This scenario fits precisely within the definition of a Marjolin ulcer, a rare but aggressive form of skin cancer arising from chronically inflamed or scarred tissue (8).

Marjolin ulcer is commonly misdiagnosed, leading to diagnostic delays. Based on the time from injury to malignant transformation, it can be divided into acute and chronic forms, with chronic lesions more frequently presenting as squamous cell carcinoma. Lesion sites most commonly involve the lower limbs (53.3%), followed by the upper limbs (18.7%), trunk (12.4%), and face/neck (5.8%) (8). Chronic inflammation plays a central role in this carcinogenic process (1).

Similar diagnostic challenges are well-established in squamous cell carcinoma of the nail unit (SCCNU), in which persistent inflammation or trauma involving the nail bed can mimic benign conditions and cause significant treatment delays (9).

Given this background, the present study systematically examines the clinical features, diagnostic pitfalls, and therapeutic dilemmas of Marjolin ulcer-type cSCC arising from chronic limb wounds and explores the underlying “chronic inflammation-to-carcinogenesis” mechanism, aiming to provide practical warning signals for clinicians.

## Case report

### Case 1

An 85-year-old man presented with an infected ingrown nail on the left thumb, which had progressively worsened over 8 months despite self-treatment. The affected area developed a persistent ulcer with swelling and pain. His past medical history was unremarkable. He had no known family history of skin cancer or chronic wounds.

#### Physical examination

**Figure 1:**
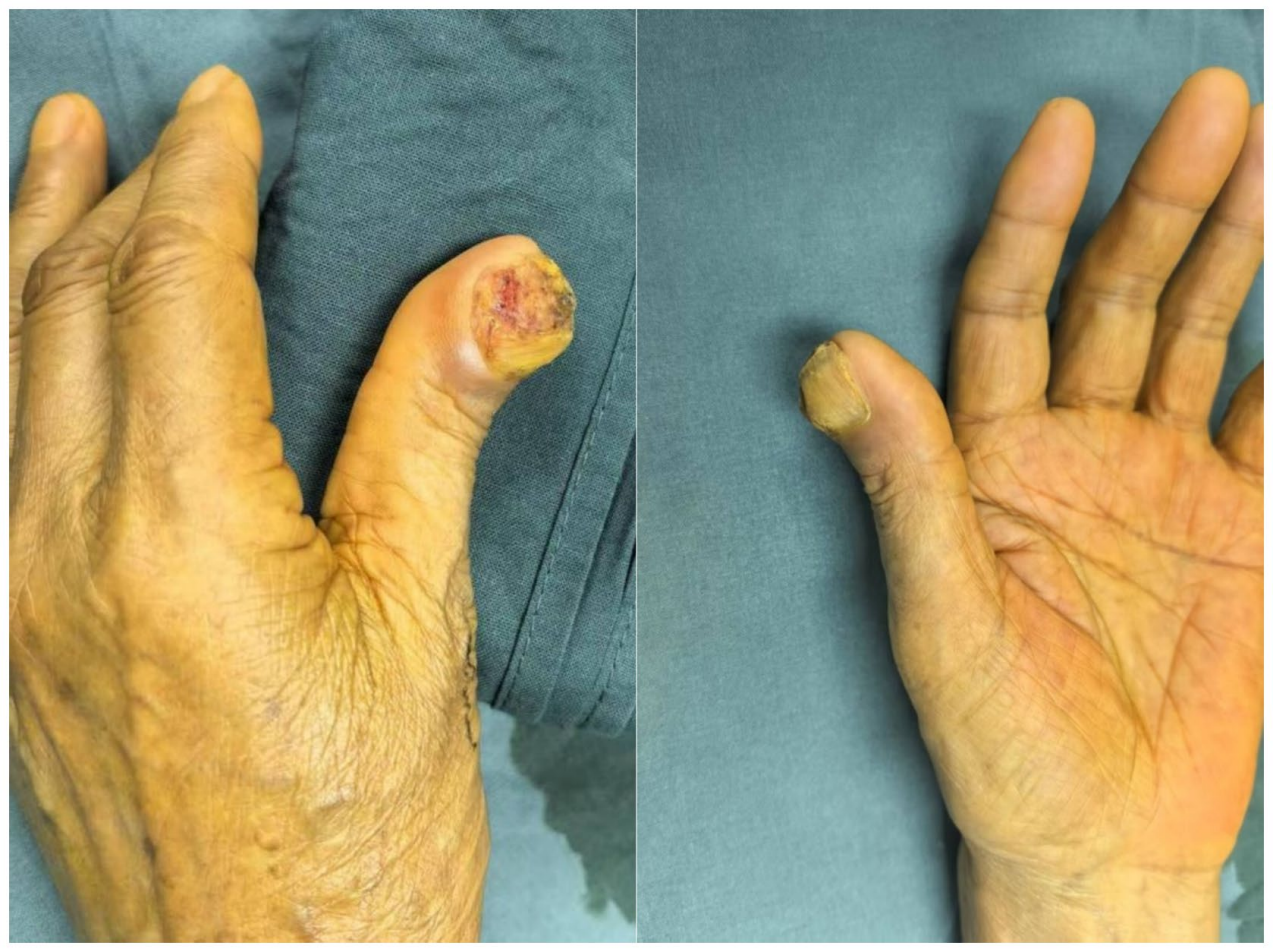
Preoperative clinical photograph of the left thumb. The affected thumb shows paronychia with inflammatory granulation tissue and nail plate deformity.

On examination, inflammatory granulation tissue and nail plate deformity were observed (Figure 1). Peripheral sensory loss was noted over the affected thumb. No regional lymphadenopathy was palpated in the axillary or epitrochlear regions. Distal perfusion was intact, with a palpable radial pulse and normal capillary refill.

#### Diagnostic workup and timeline

**Figure 2:**
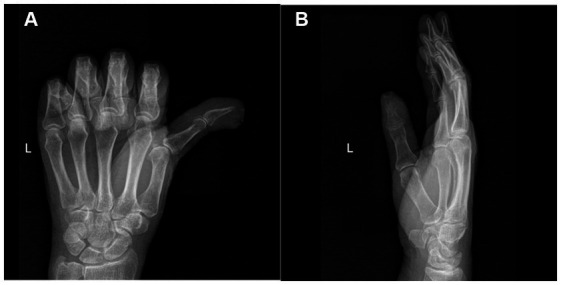
Preoperative X-ray of the left thumb. **(A)** Anteroposterior and **(B)** lateral views demonstrating osteophyte formation, decreased bone density, and sparse trabeculae in the proximal phalanx, with irregular soft tissue swelling. No definite fracture or joint space narrowing is observed.

Preoperative X-ray imaging of the left hand revealed osteophyte formation, decreased bone density, and sparse trabeculae in the proximal phalanx, with irregular swelling of the surrounding soft tissues (Figures 2A,B). No preoperative biopsy was performed, as the clinical presentation was initially attributed to chronic paronychia with secondary osteomyelitis.

**Figure 3:**
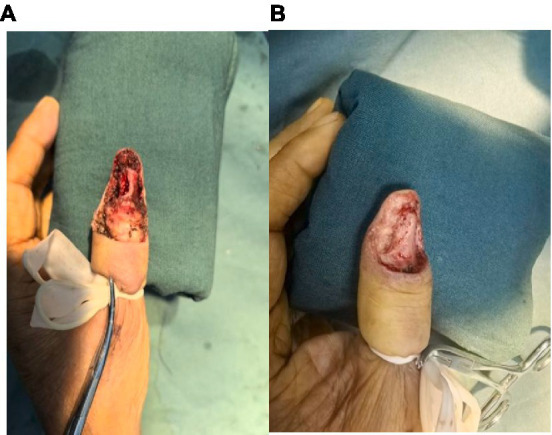
Intraoperative photographs of the left thumb. **(A)** Ill-defined tumor mass (arrows) invading the distal phalangeal bone. **(B)** Surgical defect following extended resection and curettage of the involved bone.

The initial surgical plan was tumor excision with a 1-cm margin, with the aim of preserving the finger. However, intraoperative findings revealed an ill-defined mass surrounding the distal phalanx and invading the ulnar aspect of the dorsal bone (Figure 3A). An extended resection with an additional 0.5-cm margin was performed, and the involved bone was removed using bone forceps and curettes, with the medullary cavity scraped out (Figure 3B). Intraoperative frozen-section pathology confirmed moderately differentiated squamous cell carcinoma with gross bone invasion located at the base of the nail. To achieve a negative margin while preserving as much tissue as possible, further extensive resection was performed. Postoperative permanent pathology confirmed moderately differentiated squamous cell carcinoma without evidence of lymphatic or neural invasion.

#### Clinical decision-making and follow-up

**Figure 4:**
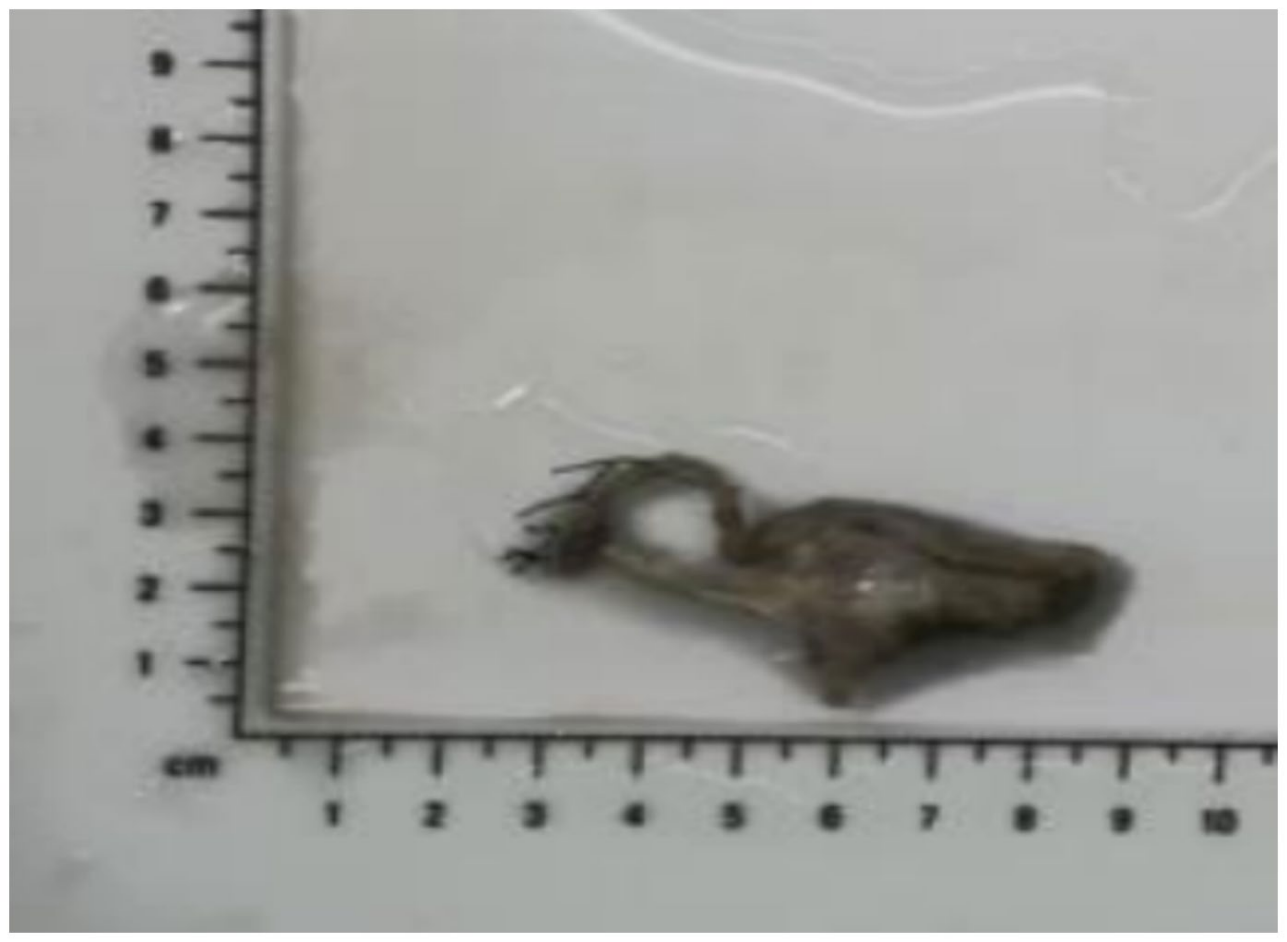
Gross specimen from the second-stage amputation of the left thumb. The specimen measures 5.5 × 3 × 0.6 cm and includes an attached skin flap (5.5 × 0.2 cm). A calcified area (2.5 × 1.5 cm) is visible within the gray–white, firm subcutaneous tissue.

**Figure 5:**
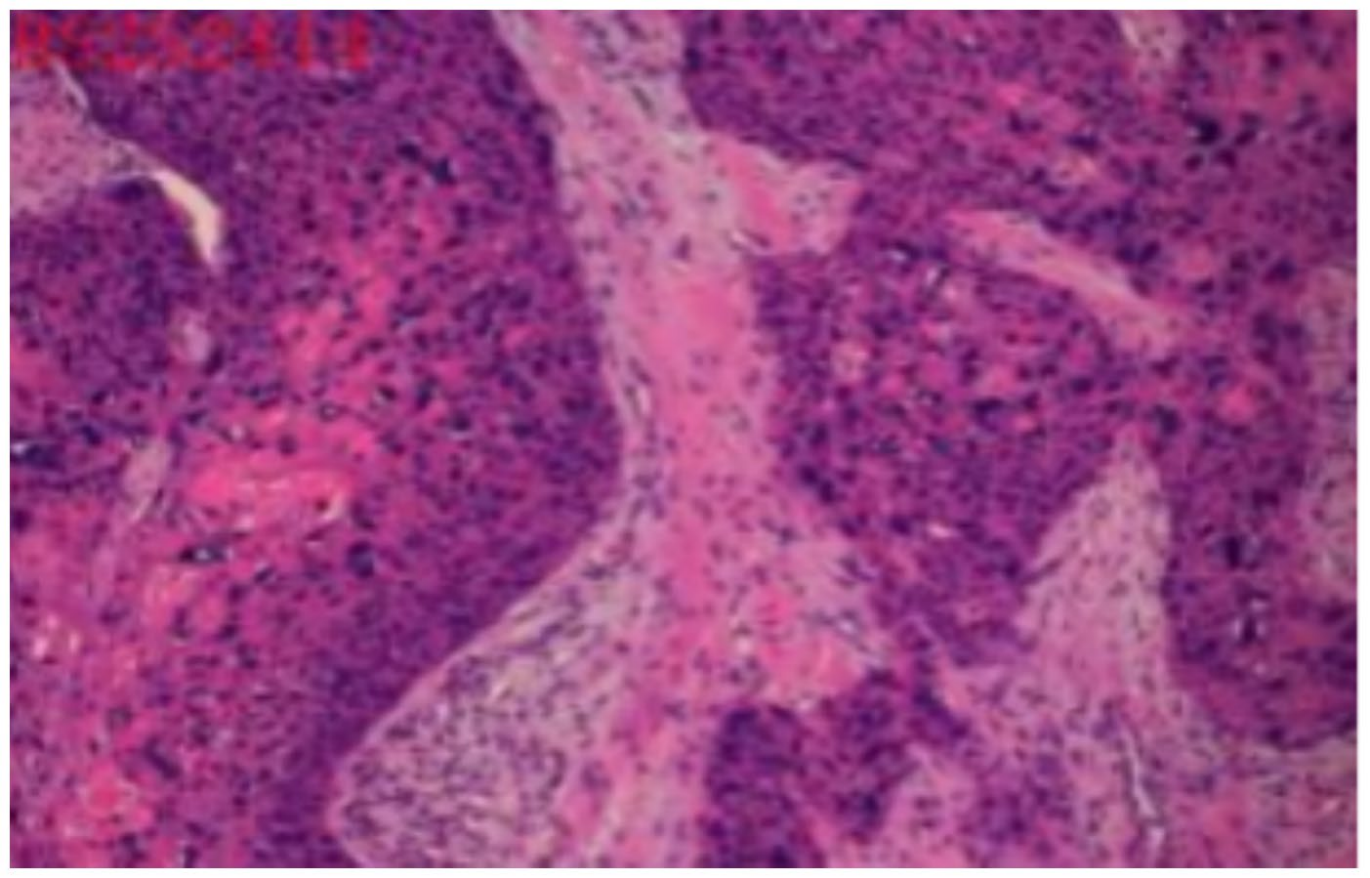
Histopathological examination of the left thumb tumor (H&E staining, original magnification ×100). Dense nests of moderately differentiated squamous cell carcinoma infiltrating the surrounding fibrovascular stroma. The pale eosinophilic areas between tumor nests represent stromal tissue. No definite keratinization or horn pearl formation is observed.

The decision-making in this case illustrates several key challenges. Initial preservation of the finger was attempted because the lesion grossly resembled that of a benign chronic paronychia. Preoperative X-ray findings of osteophyte formation and decreased bone density raised some concerns; however, the patient initially declined amputation. Intraoperatively, the discovery of an ill-defined mass invading the bone, combined with frozen section confirmation of SCC with gross bone invasion, indicated a high risk of residual disease. The surgical team discussed these findings with the patient and his family. After repeated discussions, the patient ultimately consented to amputation, which was performed at the interphalangeal joint. The specimen measured 5.5 × 3 × 0.6 cm, including an attached skin flap (5.5 × 0.2 cm). Subcutaneous tissue was gray–white and firm, containing a 2.5 × 1.5-cm calcified area (Figure 4). Histopathological examination confirmed the typical structure of squamous cell carcinoma (Figure 5, hematoxylin and eosin [H&E] staining).

A lymph node assessment was performed by clinical palpation. At the 3-month postoperative follow-up, the amputation site had healed well, and no local recurrence was observed. The patient has been advised to continue long-term surveillance.

### Case 2

A 78-year-old man presented with a non-healing ulcer on the right little finger, which had persisted for 7 years. The problem began after the removal of a “splinter” and was characterized by persistent redness, swelling, pain, and exudation. Despite multiple courses of antibiotics and exclusion of fungal infection, the ulcer failed to heal. His past medical history included atrial fibrillation controlled with aspirin, four untreated epileptic seizures, well-controlled hyperthyroidism, and a prior hernia repair performed 15 years earlier. He had no known family history of skin cancer.

#### Physical examination

A physical examination revealed inflammation of the nail folds, nail plate deformity and separation, and purulent exudation. In contrast to Case 1, no significant peripheral sensory impairment was detected. No regional lymphadenopathy was palpated. Distal perfusion was intact.

#### Diagnostic workup and timeline

**Figure 6:**
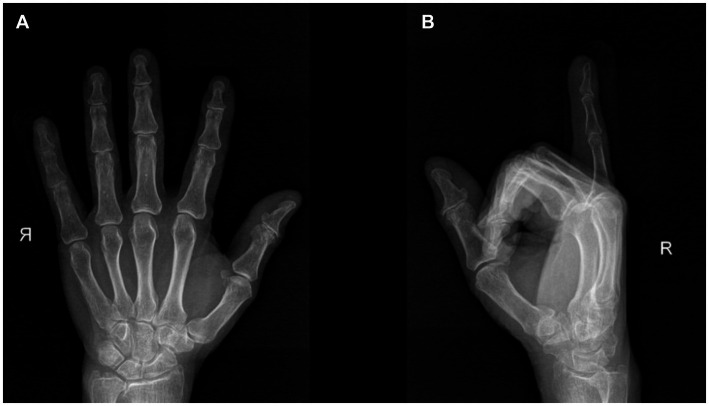
Preoperative X-ray of the right hand. **(A)** Anteroposterior and **(B)** lateral views showing normal bone architecture, good joint alignment, and soft tissue swelling around the right little finger. No fracture or bone destruction can be observed.

Preoperative X-ray imaging of the right hand showed normal bone architecture, no obvious fracture or separation, good joint alignment, and soft tissue swelling around the right little finger (Figures 6A,B). Over the 7-year course, the lesion had been repeatedly diagnosed and treated as a common infection without resolution. No preoperative biopsy was performed.

Intraoperatively, a diagnostic biopsy was performed due to the refractory nature of the ulcer and the nail plate deformity. Frozen section pathology confirmed squamous cell carcinoma. Based on this definitive diagnosis, a “right little finger amputation” was performed in the same operative session.

**Figure 7:**
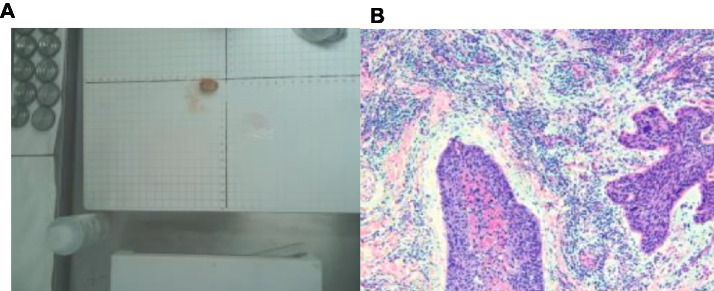
Amputated right little finger specimen and histopathology. **(A)** Gross specimen showing an ulcer measuring 1.3 × 0.7 cm (arrow). **(B)** Histopathological examination (H&E staining, original magnification ×100) confirming moderately differentiated squamous cell carcinoma. The tumor forms irregular nests with infiltrative growth into the surrounding stroma. No definite keratin pearls, lymphovascular invasion, or perineural invasion are identified.

The amputated specimen (Figure 7A) showed a 1.3 × 0.7-cm gray–red ulcer with a firm texture, which covered but did not invade the bone. Permanent pathological examination confirmed moderately differentiated squamous cell carcinoma with an infiltration depth of 0.1 cm, without lymphatic or neural invasion (Figure 7B, H&E staining).

#### Clinical decision-making and follow-up

This case highlights the diagnostic pitfall of attributing a chronic, non-healing wound to a persistent infection. Despite multiple courses of antibiotics and exclusion of fungal infection for more than 7 years, malignancy was not initially suspected. The eventual decision to perform an intraoperative diagnostic biopsy—prompted by the refractory nature of the ulcer—led to the definitive diagnosis. The patient was informed of the intraoperative biopsy findings and consented to the amputation.

In contrast to Case 1, the absence of bone invasion and the very superficial infiltration depth (0.1 cm) suggest that an earlier biopsy might have permitted a less extensive, finger-sparing resection. Preoperative imaging (X-ray) showed no bone abnormalities. A lymph node assessment was performed by clinical palpation.

At the 3-month follow-up, the amputation site remained well healed, and no recurrence was detected. Long-term surveillance has been recommended.

### Case 3

A 70-year-old man was admitted for a long-standing, non-healing ulcer on the right plantar surface with purulent discharge. The ulcer developed at the weight-bearing heel region following open reduction and internal fixation for a calcaneal fracture and splenectomy for a splenic rupture 30 years earlier. The ulcer, which was initially coin-sized, gradually enlarged despite long-term self-care. His past medical history included the aforementioned trauma. He had no known family history of skin cancer.

#### Physical examination

On examination, a large chronic ulcer approximately 12 × 10 cm was found extending to the calcaneus, with inflammatory granulation tissue and purulent discharge. The distal ends of the toes on the right foot had decreased sensation and reduced pinprick sensation. Blood supply was good, with a palpable dorsalis pedis pulse. No regional inguinal lymphadenopathy was palpated.

#### Diagnostic workup and timeline

**Figure 8:**
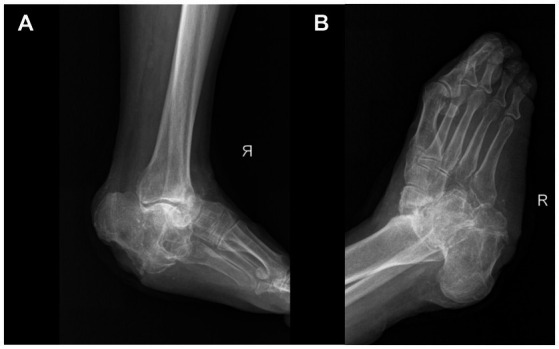
Preoperative imaging of the right foot and ankle. **(A)** X-ray and **(B)** CT scan demonstrating decreased bone density, sparse trabeculation, osteophyte formation with marginal sclerosis, and obliteration of the right calcaneocuboid joint space.

Comprehensive preoperative imaging was performed. X-ray, computed tomography (CT), and magnetic resonance imaging (MRI) of the right foot and ankle revealed decreased bone density, sparse trabeculation, osteophyte formation, and sclerosis at the joint margins and loss of the right calcaneocuboid joint space (Figures 8A,B). Despite these findings suggestive of deep tissue involvement, the clinical diagnosis remained “chronic ulcer of the right plantar surface,” and malignancy was not suspected.

The patient was informed preoperatively that the extent of surgery would depend on intraoperative findings and pathological results and that amputation was a possibility. The initial surgery was a “right foot chronic ulcer repair” with complete excision and a 1-cm margin. No preoperative biopsy was performed.

Postoperative pathological examination unexpectedly revealed moderately differentiated SCC invading the calcaneus, with a maximum infiltration depth of 1.1 cm. All margins were clear; however, the tumor was only 1 mm from the deep margin. Due to extensive local infiltration and bone invasion, radical local excision was not possible. Based on the prior preoperative discussion regarding possible amputation, the patient accepted a second-stage below-knee amputation.

**Figure 9:**
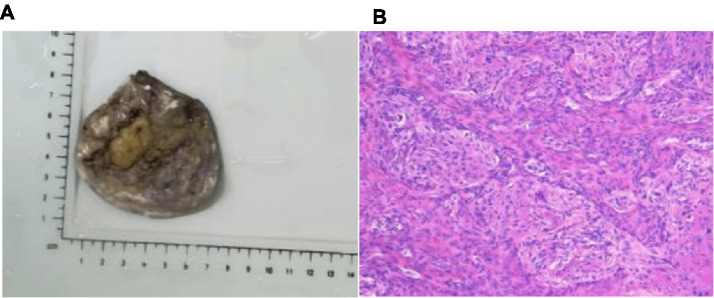
Below-knee amputation specimen and histopathology. **(A)** Gross specimen showing a large ulcerated area measuring approximately 12 × 10 cm involving the plantar heel. **(B)** Histopathological examination (H&E staining, original magnification ×100) revealing moderately differentiated squamous cell carcinoma. Tumor cells form cord-like and nested structures with marked nuclear atypia and infiltrative growth into the eosinophilic stroma. A tendency toward squamous differentiation is evident.

The amputated specimen is shown in Figure 9A. Permanent pathological examination confirmed moderately differentiated SCC with a deepest interstitial infiltration depth of 1.1 cm, no evidence of lymphatic or neural invasion, and clear margins (Figure 9B, H&E staining).

#### Clinical decision-making and follow-up

This case demonstrates the catastrophic consequences of decades-long diagnostic delay. The lesion was clinically managed as a “chronic ulcer” for 30 years, during which it progressively enlarged from the size of a coin to 12 × 10 cm and deeply invaded the calcaneus. Preoperative imaging had already revealed bone involvement and joint space obliteration, yet the clinical suspicion of malignancy remained low. The first operation was planned as a benign ulcer repair, and malignancy was only discovered on postoperative pathology. The deep margin was merely 1 mm from the tumor, necessitating a below-knee amputation. Importantly, as the possibility of amputation had been discussed with the patient preoperatively, he was able to accept this outcome.

A lymph node assessment was performed by clinical palpation. At the 3-month follow-up after below-knee amputation, the stump had healed uneventfully, and no local recurrence was found. Long-term surveillance is ongoing.

### Case 4

A 59-year-old man with a 10-year smoking history presented with a mass on the right lower limb, which had been noticed for more than 1 year. The mass appeared at the site of an abrasion caused by a bicycle accident approximately 2 years prior. His past medical history was otherwise unremarkable. He had no known family history of skin cancer.

#### Physical examination

A physical examination revealed a round mass measuring approximately 3 × 2 cm in the right popliteal fossa, with an irregular surface and localized tenderness (+). Sensation and pinprick sensation of the right lower limb were comparable to the contralateral side. The dorsalis pedis artery pulse was palpable, with good distal blood supply. No regional inguinal lymphadenopathy was palpated.

#### Diagnostic workup and timeline

Preoperative imaging details were not specified in the available clinical records. Based on the clinical examination findings of a cauliflower-like mass with an irregular surface and firm texture, the clinical diagnosis was “right leg mass.” No preoperative biopsy was performed.

An “enlarged excision” was performed with a 5-mm margin from the edge of the mass, removing tissue measuring 3 × 2.9 × 0.7 cm, with an attached skin flap of 3 × 2.9 cm. Intraoperatively, the mass was noted to have a cauliflower-like appearance with visible inflammatory granulation tissue. The cut surface showed slightly firm gray–white tissue.

**Figure 10:**
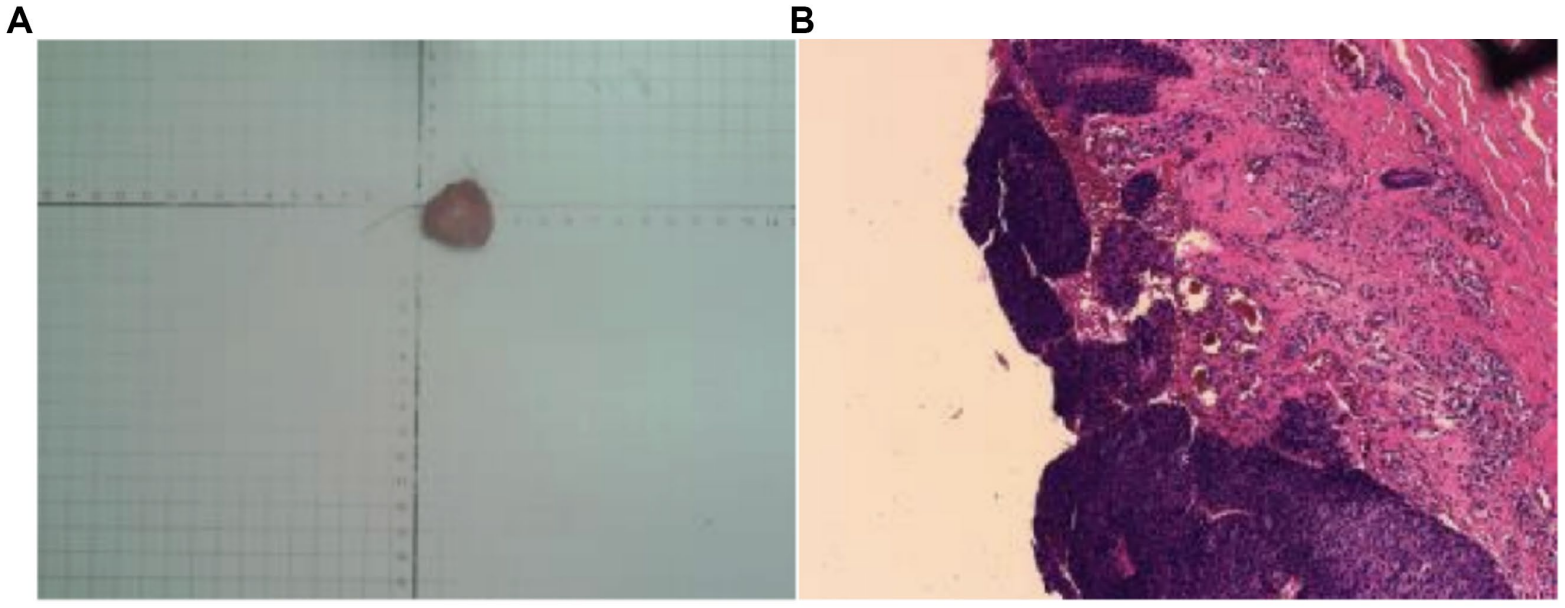
Right lower limb mass specimen and histopathology. **(A)** Gross specimen from the enlarged excision of the right popliteal fossa mass, measuring 3 × 2.9 × 0.7 cm. **(B)** Histopathological examination (H&E staining, original magnification ×100) showing squamous cell carcinoma *in situ*. Abnormally proliferating squamous epithelial cells form irregular nests confined within the epithelium. Cellular atypia and architectural disorganization are evident, without breach of the basement membrane.

Permanent pathological examination confirmed squamous cell carcinoma *in situ* (Figure 10, H&E staining). No lymphatic or neural invasion was identified.

#### Clinical decision-making and follow-up

In contrast to the other three cases, this lesion presented as a mass rather than an ulcer, with a relatively short interval of approximately 2 years from the initial injury to clinical presentation. The decision to perform an enlarged excision with a 5-mm margin, rather than a simple excision, suggests that the surgeon had a degree of preoperative suspicion based on the cauliflower-like appearance and firm texture. The patient underwent the planned procedure without any specific preoperative concerns.

The diagnosis of SCC *in situ* carries a favorable prognosis compared with invasive SCC. A lymph node assessment was performed by clinical palpation. At the 3-month follow-up, the excision site showed good healing without evidence of recurrence. Long-term surveillance has been recommended.

A summary of patient demographics, clinical characteristics, and surgical management is provided in Table 1.

**Table 1:** Summary of patient demographics, clinical course, surgical management, and follow-up.

Case	Age Sex	Affected area	Trigger	Chronic wound duration	Bone invasion	Final surgical management
1	85 Male	Left thumb	Infected ingrown nail	8 months	Yes	Amputation of the left thumb
2	78 Male	Right little finger	Splinter	7 years	No	Amputation of the right little finger
3	70 Male	Right plantar heel	Post-surgical ulcer (calcaneal fracture)	30 years	Yes	Below-knee amputation
4	59 Male	Right popliteal fossa	Bicycle abrasion	2 years	No	Wide local excision

## Discussion

A key strength of this case series lies in its heterogeneity. The four cases differed markedly in anatomical site (thumb, little finger, plantar heel, and popliteal fossa), initial trigger (ingrown nail, splinter, post-surgical scar from calcaneal fracture, and abrasion from a bicycle accident), and chronicity of the wound (ranging from 8 months to 30 years). Despite this diversity, a common clinical thread unites them: Any long-standing, seemingly benign chronic wound on the limbs, regardless of its initial cause, may harbor the potential for malignant transformation and ultimately lead to devastating consequences, including amputation. This heterogeneity, rather than weakening the series, reinforces the generalizability of its core message—chronic wounds should never be dismissed as benign without thorough evaluation.

Accordingly, all four patients were followed up after surgery. At the most recent follow-up (3 months postoperatively), no local recurrence had been observed in any case. Long-term follow-up is ongoing. Regular clinical follow-up is essential for patients with cSCC arising from chronic wounds, given the aggressive nature of Marjolin ulcer. Examinations should include inspection and palpation of the resected site and regional lymph nodes, with the frequency and use of imaging tailored to individual risk factors. The short follow-up period in this series represents a limitation, and continued surveillance is necessary to fully characterize the long-term oncologic outcomes.

### Warning signals for high-risk chronic wounds

For general cutaneous squamous cell carcinoma (cSCC) lesions, local excision is usually sufficient. However, Marjolin ulcer is often overlooked because it is misdiagnosed as a chronic wound, leading to significant progression in cSCC staging, frequently accompanied by bone infiltration and extensive soft tissue invasion, ultimately requiring wider surgical margins or amputation. Diagnostic delays, ranging from 8 months to 30 years, result in treatment shifting from curative local excision to more extensive surgeries such as amputation, severely affecting limb function and long-term prognosis.

Based on this case series and the reviewed literature, we propose a set of practical clinical warning signals that may help identify high-risk chronic wounds and break the cycle of misdiagnosis and delay. All four cases involved elderly male patients; however, their clinical presentations were otherwise heterogeneous. Through careful observation of their commonalities, we identified three key aspects that may aid in the early recognition of cSCC in the context of a Marjolin ulcer.

First, patient characteristics and disease course can provide important baseline clues. One case showed relatively rapid wound progression in an elderly patient, suggesting that advanced age itself may serve as a warning signal. Another case had a disease course spanning several decades, allowing ample time for malignant progression within the chronic wound microenvironment. All patients were male, which is consistent with the previously reported male predominance in Marjolin ulcer.

Second, wound characteristics merit close attention. All lesions in this series presented with obvious ulcerative features, including chronic exudation despite preserved blood supply, and exhibited the characteristic granulation and scar tissue noted in Marjolin ulcers. Notably, deeper tumor progression in two cases was accompanied by significant sensory loss on physical examination, indicating that impaired sensation may signal nerve involvement by the tumor. Overall, upper limb lesions in this series appeared relatively less severe than their lower limb counterparts.

Third, pathological examination remains the definitive diagnostic tool. All four patients were eventually diagnosed with cSCC, and scar tissue was present in every case. For more advanced lesions, imaging studies revealed corresponding bone and soft tissue changes.

Taken together, these observations are summarized in Table 2. We emphasize that these signals are offered not as a standardized protocol but as a set of clinical warning signals intended to raise suspicion and prompt timely biopsy or specialist referral.

**Table 2:** Proposed clinical warning signals for high-risk chronic wounds suspicious for Marjolin ulcer.

Domain	Warning signal	Clinical significance
Time	Wound that fails to heal or recurs within 2–3 months despite appropriate care	Short-term non-healing suggests an underlying pathological process distinct from common benign wounds and warrants further investigation.
	Chronic wound persisting for decades (e.g., >10 years)	Extended duration provides a prolonged window for cumulative malignant transformation within the chronic inflammatory microenvironment.
Morphology	Cauliflower-like, stone-hard, or ulcerative growth with elevated, firm, and easily bleeding margins	These macroscopic features are classic signs of malignant transformation in chronic wounds and should trigger immediate suspicion.
	Persistent exudation, erythema, swelling, and pain, with preserved blood supply but local sensory loss	The discordance between good perfusion and sensory deficit suggests deep infiltration involving peripheral nerves, a feature of advanced disease.
Context	Elderly male patient	Consistent with the known demographic profile of Marjolin ulcer (male predominance, peak incidence in the 5th–6th decades of life).
	Lesion arising on a background of old trauma, burn scar, or long-standing non-healing ulcer.	Any chronic wound environment, regardless of the initial insult, can serve as a carcinogenic niche and should be evaluated with heightened vigilance.

Summary of practical clinical warning signals derived from the present case series and literature review. These signals are not intended as diagnostic criteria but rather as a practical aid to raise clinical suspicion and prompt timely biopsy or specialist referral.

### Diagnosis pathway of the disease

Early imaging studies help determine the tumor extent and detect occult lymph node metastases, thereby improving treatment options and prognosis. For suspected locally advanced lesions, imaging of the primary site is recommended. X-ray is the basic method for screening bone erosion. Magnetic resonance imaging (MRI) is more sensitive in assessing perineural spread, while computed tomography (CT) is more advantageous in evaluating bone invasion and deep lymph node metastases (1). Lymph node metastasis is the most common route of metastasis in squamous cell carcinoma (SCC) and significantly affects prognosis, thus requiring early assessment (10).

Pathology remains the gold standard for diagnosis. A full-thickness skin biopsy or excisional biopsy from the most representative active lesion can provide a definitive diagnosis. For atypical morphology, immunohistochemical (IHC) testing is crucial: markers such as p40, p63, and cytokeratin 5/6 (CK5/6) exhibit high sensitivity and specificity for confirming squamous differentiation (11), whereas cytokeratins (such as CK34βE12), epithelial membrane antigen (EMA), and p63 can confirm epithelial origin and squamous differentiation, and a high Ki-67 proliferation index supports the diagnosis of malignancy and may provide prognostic information regarding tumor aggressiveness (12, 13). Although the diagnosis in our four cases was established primarily on H&E morphology, the consistent histopathological features—including infiltrative nests of atypical keratinocytes, stromal desmoplasia, and, in Cases 1–3, clear evidence of invasive growth—provided sufficient diagnostic certainty. In diagnostically challenging cases where morphology alone is ambiguous, the addition of the aforementioned IHC markers is recommended to confirm squamous lineage and to exclude histologic mimickers such as basal cell carcinoma, melanoma, or poorly differentiated adenocarcinoma.

### Treatment guidelines

Complete surgical excision is the curative treatment for localized squamous cell carcinoma (SCC). The width of the resection margin has risk-stratification significance: The current view suggests that a 5-mm margin is appropriate for low-risk tumors. For high-risk cSCC, extending the margin to 6–10 mm is recommended; however, an appropriate margin size has not yet reached a consensus (7, 14). For cases where the distance to the resection margin is difficult to determine, Mohs microsurgery may be considered to maximize tissue preservation. Treatment strategies can be stratified: For primary low-risk SCC, wide excision is recommended; for primary high-risk SCC, more extensive excision or Mohs surgery is suggested; for 4–5% of patients with locally advanced or metastatic SCC, an individualized multidisciplinary treatment plan should be developed, which may combine radiotherapy and systemic therapy (14, 15). In systemic therapy, although traditional cytotoxic chemotherapy (such as platinum-based agents and 5-fluorouracil) has some efficacy, its significant toxic side effects and high recurrence rate are problematic (13, 16). Currently, immune checkpoint inhibitors targeting PD-1/PD-L1 and EGFR-targeted therapies represent transformative treatment options for advanced cSCC (16).

### Mechanism of disease occurrence

From a mechanistic perspective, the transition of chronic wounds to squamous cell carcinoma is not a passive process but an active pathological evolution driven by an abnormal wound healing microenvironment. This microenvironment is characterized by persistent inflammation (e.g., release of interleukin 6 [IL-6], HMGB1, and other pro-inflammatory mediators), progressive fibrosis with matrix stiffening (driven by transforming growth factor-*β* [TGF-β]) (16), and impaired local immune surveillance (17). Disruption of the wound repair process leads to a state of chronic non-healing, which, in turn, can promote skin tumorigenesis—a phenomenon in which tumors effectively “hijack” the wound healing response to enhance their survival and growth (18). In this context, keratinocytes face endogenous mutagenic pressure and accumulate mutations in TP53- and NOTCH-driven genes under the influence of continuous proliferative signals, eventually acquiring an invasive phenotype (17).

In the four cases presented in this study, several of these mechanisms were clinically evident. In Cases 1, 2, and 3, the wounds exhibited classic features of chronic inflammation—persistent exudation, granulation tissue formation, and recurrent cycles of partial healing and breakdown—suggesting sustained exposure to inflammatory cytokines. Case 3, with a 30-year disease course, represents the most extreme example: The prolonged duration allowed cumulative malignant transformation within the chronic inflammatory microenvironment, consistent with the principle that, the longer a chronic wound persists, the higher the risk of Marjolin ulcer development (17). Case 4, which presented as a mass rather than an ulcer, may reflect a different stage along the inflammation-fibrosis-genomic instability axis, in which the proliferative phase of wound healing had already given way to tumor formation with minimal residual ulceration. In Cases 1 and 3, the presence of calcified areas and bone invasion on imaging provided objective evidence of chronic osteomyelitis, a known contributing factor to malignant transformation via oxidative stress and sustained inflammatory signaling (17). Taken together, these four cases illustrate how the malignant cycle of inflammation-fibrosis-genomic instability, operating within a wound microenvironment characterized by impaired immune surveillance, can drive the progression from a benign chronic wound to an aggressive Marjolin ulcer.

## Data Availability

The original contributions presented in the study are included in the article/supplementary material, further inquiries can be directed to the corresponding author.
